# Impact of Alkanediols on Stratum Corneum Lipids and Triamcinolone Acetonide Skin Penetration

**DOI:** 10.3390/pharmaceutics13091451

**Published:** 2021-09-11

**Authors:** Melanie Sigg, Rolf Daniels

**Affiliations:** Department of Pharmaceutical Technology, Eberhard Karls University, Auf der Morgenstelle 8, 72076 Tuebingen, Germany; melanie.sigg@uni-tuebingen.de

**Keywords:** alkanediols, preservation, triamcinolone acetonide, ex vivo skin penetration, stratum corneum lipids, confocal Raman spectroscopy (CRS)

## Abstract

Alkanediols are widely used as multifunctional ingredients in dermal formulations. In addition to their preservative effect, considering their possible impact on drug penetration is also essential for their use. In the present study, the influence of 2-methyl-2,4-pentanediol, 1,2-pentanediol, 1,2-hexanediol and 1,2-octanediol on the skin penetration of triamcinolone acetonide from four different semisolid formulations was investigated. Furthermore, confocal Raman spectroscopy measurements were performed to examine the influence of the alkanediols on stratum corneum lipid content and order. Alkanediols were found to increase the penetration of triamcinolone acetonide. However, the extent depends strongly on the formulation used. In certain formulations, 1,2-pentanediol showed the highest effect, while in others the penetration-enhancing effect increased with the alkyl chain length of the alkanediol used. None of the tested alkanediols extracted lipids from the stratum corneum nor reduced its thickness. Notwithstanding the above, the longer-chained alkanediols cause the lipids to be converted to a more disordered state, which favors drug penetration. This behavior could not be detected for the shorter-chained alkanediols. Therefore, their penetration-enhancing effect is supposed to be related to an interaction with the hydrophilic regions of the stratum corneum.

## 1. Introduction

Many preservatives used in formulations for dermal application have fallen into disrepute, or have even been misreported because of undesirable effects, e.g., endocrine disrupting properties, during the last decade. Therefore, the demand for alternative preservatives is steadily increasing [1,2,3]. Alkanediols are promising candidates for this purpose. The most commonly used alkanediols are 1,2-pentanediol, 2-methyl-2,4-pentanediol (hexylene glycol), 1,2-hexanediol and 1,2-octanediol. Their preservative effect has already been demonstrated [4,5] and can primarily be attributed to their amphiphilic properties. With their hydrophilic head group on the one hand and their lipophilic alkyl chain on the other hand, they are enabled to interact and disorder the cell membranes of microorganisms [5]. Due to their amphiphilic nature, they can also interact with the surfactant and co-surfactant system of an emulsion or cream. This interaction becomes more intense with rising alkyl chain length [4,6]. In addition, the alkanediols used for alternative preservation must be safe and non-hazardous to health. The Cosmetic Ingredient Review Expert Panel rates the application of the alkanediols examined in our study as safe when used in the recommended concentration range [7,8]. Besides their preservative effect, they are known as multifunctional excipients exhibiting moisturizing and solubilizing properties [9,10].

Moreover, alkanediols are reported to act as penetration enhancers [11,12]. This is a particularly important aspect to be considered when using alkanediols in topical drug formulations. Therefore, the present study focused on this effect. The answer to this question is of paramount importance when an existing formulation is modified by substituting alkanediols for a conventional preservative. In accordance with the European Medicines Agency (EMA) Draft Guideline on quality and equivalence of topical products the impact on the dermal pharmacokinetic has to be assessed [13,14].

Triamcinolone acetonide (TAA), the model drug, is a glucocorticoid, that is widely used for treating inflammatory, allergic and pruritic dermatoses [15,16]. The glucocorticoid receptors which have to be addressed are located in the epidermal and dermal cells [17]. Therefore, after applying the formulation to the skin, TAA has firstly to overcome the stratum corneum (SC), which is the outermost permeability barrier of the skin, to reach its target structures. To this end, TAA is a perfectly suitable model drug for studying the influence of alkanediols on skin penetration.

Primarily, the penetration behavior of TAA as the active pharmaceutical ingredient (API) in different formulations and the effect of incorporating alkanediols was examined. Secondly, the impact of alkanediols on the lipid structures of the SC was investigated using confocal Raman spectroscopy (CRS).

As bases, we selected the same formulations as in the previous study focusing on the release of TAA [18], namely the nonionic hydrophilic cream (NHC), the anionic hydrophilic cream (AHC), and the aqueous carbomer gel (ACG) according to the German Pharmacopeia (DAB) [19] as well as the basic cream (BC) according to the German Drug Codex DAC (DAC) [20].

## 2. Materials and Methods

### 2.1. Materials

1,2-Pentanediol, 2-methyl-2,4-pentanediol, and 1,2-hexanediol (Sigma-Aldrich Chemie GmbH, Taufkirchen, Germany), polysorbate 60 (Kolliphor PS 60), cetostearyl alcohol (Kolliwax CSA), cetyl alcohol (Kolliwax CA), emulsifying cetostearyl alcohol type A (Kolliphor CSA) (BASF SE, Ludwigshafen, Germany), glycerol 85%, propylene glycol (Dr. Willmar Schwabe GmbH & Co. KG, Karlsruhe, Germany), white soft paraffin (Sasol Performance Chemicals, Hamburg, Germany), liquid paraffin (Hansen & Rosenthal KG, Hamburg, Germany), carbomer 50,000 (Fagron GmbH & Co. KG, Glinde, Germany), sodium hydroxide (Chemical supply of the University, Tuebingen, Germany), medium-chain triglycerides (Evonik Industries AG, Essen, Germany), glycerol monostearate 60, macrogol 20 glycerol monostearate, disodium hydrogen phosphate, potassium dihydrogen phosphate, sodium chloride, triamcinolone acetonide (micronized) (Caesar & Loretz GmbH, Hilden, Germany), bovine serum albumin (Carl Roth GmbH & Co. KG, Karlsruhe, Germany), acetonitril HPLC gradient grade (Honeywell Specialty Chemicals Seelze GmbH, Seelze, Germany), PTFE filters (pore size: 0.2 µm, diameter: 25 mm, Chromafil Xtra H-PTFE-20/25, Macherey-Nagel GmbH & Co. KG, Dueren, Germany), Neg-50 Frozen Section Medium (Thermo Fisher Scientific Inc., Waltham, MA, USA). 1,2-Octanediol (dermosoft Octiol) was kindly donated by Evonik Dr. Straetmans GmbH, Hamburg, Germany.

### 2.2. Preparation of the Test Formulations

Table 1 provides an overview over the compositions of the compendial formulations examined in the present study. From these bases, the test formulations were prepared by adding 0.1% TAA, representing the upper recommended usage concentration [16]. In addition, all preparations were produced with an addition of 5% of the respective alkanediol. The amount of alkanediol added was deducted from the water amount. The reference BC contains 10% propylene glycol, which is likewise an alkanediol. Thus, 10% propylene glycol were substituted with 5% of the examined alkanediols to isolate the effect of the studied alkanediols from that of propylene glycol. The remaining 5% were replaced by water. The detailed manufacturing processes of each formulation are described in Sigg and Daniels [18].

### 2.3. Dermatomed Pig Ear Skin

Porcine ear skin was used for the penetration experiments because it is similar to the human skin in terms of histology and morphology [21,22,23]. The ears used for the experiments were provided by either a local butcher (Bio Metzgerei Griesshaber, Moessingen-Oeschingen, Germany) or by the University Hospital Tuebingen (Department of Experimental Medicine, Tuebingen, Germany). The Institute of Pharmaceutical Technology is registered for the use of animal products with the Tuebingen District Office (registration number: DE 08 4161052 21, approval date: 22 December 2015). Prior to and after cutting the full-thickness skin from the cartilage, it was cleaned with isotonic saline and cotton swabs. The skin was cut into strips approximately 4 cm wide and fixed with pins on a Styrofoam plate wrapped with aluminum foil. The hair was trimmed to approximately 0.5 mm using a hair clipper (QC5115/15, Philips, Amsterdam, The Netherlands). After cutting the skin to a thickness of 1 mm with a dermatome (GA 630, Aesculap AG & Co. KG, Tuttlingen, Germany), circles with a diameter of 25 mm were punched out from the prepared strips. Subsequently, the skin punches were wrapped in aluminum foil and stored at −30 °C until the day of the experiment.

### 2.4. Ex Vivo Penetration Studies

Ex vivo penetration studies were performed with modified Franz diffusion cells (Gauer Glas, Puettlingen, Germany), having a receptor volume of 12 mL as well as an inner diameter of 15 mm, representing an actual penetration area of 1.77 cm².

Owing to its high lipophilicity, the solubility of TAA in phosphate-buffered saline (PBS) is not high enough to achieve sink conditions in the experiments presented below [18]. Therefore, to increase the TAA solubility, 4% (*w*/*w*) bovine serum albumin (BSA) was added to PBS. The acceptor medium was warmed to 32 °C, representing the skin surface temperature, and stirred at a speed of 500 rpm. The Franz cells were equipped with dermatomed porcine skin. Approximately 15 mg of the formulation were applied to the skin after an equilibrium period of 30 min to work under finite dosing conditions [24,25]. The formulation was evenly distributed on the skin by means of a plexiglas cylinder and the mass of the applied preparation was determined accurately. 

An incubation period of 24 h was chosen, as TAA is usually applied only once per day because of its depot effect [26,27]. Thereafter, the skin samples were detached from the Franz diffusion cells. The formulation remaining on the skin surface was wiped away with a cotton swab soaked with 500 μL of isotonic saline. After the penetration area (diameter: 15 mm) has been punched out, the skin pieces were weighted and immediately frozen in aluminum molds in liquid nitrogen to prevent further penetration.

The dissection of the skin was carried out by means of a cryo-microtome (HM 560 Cryo-Star; Thermo Fisher Scientific Inc., Waltham, MA, USA). After the frozen skin samples were fixed to the sample holder using a frozen section medium (NEG 50; Thermo Fisher Scientific Inc., Waltham, MA, USA), the skin was cut into slices having a thickness of 16 µm. The first incomplete as well as the first complete section are assigned to the SC. The following 14 sections represent the living epidermis. The last sections belong to the dermis [28,29,30]. The segments were collected and extracted with 1 mL acetonitrile. After vortexing (Vortex 2, IKA-Werke GmbH & Co. KG, Staufen, Germany) for 10 s, the samples were further extracted in an ultrasonic bath for 30 min. The extraction medium was then withdrawn and filtered through a 0.2 μm PTFE filter (Chromafil Xtra H-PTFE-20/25, Macherey-Nagel, Dueren, Germany). The TAA content was analyzed by HPLC (high-performance liquid chromatography) as described below.

The cotton swab used for wiping the remaining formulation was extracted with 9.5 mL of acetonitrile by vortexing for 1 min, sonication for 30 min and subsequently vortexing again for 1 min. The HPLC analysis was performed after filtration through a 0.2 µm PTFE filter (Chromafil Xtra H-PTFE-20/25, Macherey-Nagel, Dueren, Germany).

Formulation residues that stick to the top of the Franz cells were removed with a second cotton swab. This swab was extracted with the same extraction method used for the first cotton swab. The determined API amount detected on the second cotton swab was then subtracted from the initially applied amount yielding the final amount of API applied to the skin.

Samples were also taken from the acceptor medium to determine permeated TAA. Acetonitrile was added to the sample (3 + 1) to remove BSA prior to HPLC analysis [31]. Following 10 s of vortexing (Vortex 2, IKA-Werke GmbH & Co. KG, Staufen, Germany), samples were centrifuged (MiniSpin, Eppendorf AG, Hamburg, Germany) at 13,400 rpm for 15 min to pellet BSA. Subsequently, an HPLC analysis of the clear supernatant was performed.

Experiments were performed in triplicate.

### 2.5. HPLC Analysis

The different TAA solutions were analyzed using an HPLC system (LC 20AT, DGU 20A5R, SIL 20AC HAT, CTO 10ASVP, CBM 20 A, Shimadzu GmbH, Duisburg, Germany) in combination with an UV detector (SPD 20A, Shimadzu GmbH, Duisburg, Germany). For TAA quantification, the separation was conducted at 30 °C on a RP-18 column (Nucleosil 100-5 C18 125/4, Macherey-Nagel GmbH & Co. KG, Dueren, Germany). The mobile phase was acetonitrile-water (40:60, *v:v*). The flow rate was adjusted to 0.8 mL/min, and 20 µL sample volume was injected. UV absorption was determined at a wavelength of 254 nm. The TAA retention time was 4.8 min. 

The limit of quantification (LOQ) was calculated according to the ICH (International Council for Harmonisation of Technical Requirements for Pharmaceuticals for Human Use) guidelines [32]. The LOQ for the permeation samples was 0.2406 µg/mL and for the penetration samples 0.0254 µg/mL, respectively.

### 2.6. Calculation of the Penetrated TAA Amount

The calculation of the penetrated TAA amount is normalized to an applied amount of 15 mg formulation as well as to a mass of the skin piece of 175 mg. Further, the penetrated TAA mass is averaged across the overall punched out skin piece. Although not explicitly indicated all penetration experiments are referred to the same time interval.

The quantity of TAA penetrating into the skin piece per unit area was calculated according to Equation (1).

(1) $$m\left(\mathrm{TAA}\right)\left[\frac{\mathrm{ng}}{{\mathrm{cm}}^{2}}\right]=\sum \frac{c\left(\mathrm{TAA}\right)\cdot V\left(\mathrm{acetonitrile}\right)\cdot 15\;\mathrm{mg}\;\cdot \;175\;\mathrm{mg}}{m\left(\mathrm{formulation}\right)\cdot m\left(\mathrm{total}\;\mathrm{skin}\;\mathrm{piece}\right)\cdot 1.77\;{\mathrm{cm}}^{2}}$$

where in c(TAA) is the concentration of TAA determined by HPLC, V(acetonitrile) is the volume of acetonitrile used to extract the skin segments, m(formulation) indicates the exact mass of preparation applied and m(total skin piece) denotes the accurate mass of the respective total skin piece. The calculation was performed separately for SC, epidermis and dermis and the cumulative amount of penetrated TAA was assessed by summing up data from all three layers.

Accordingly, the penetrated fraction of TAA relative to the totally applied TAA mass was calculated according to Equation (2).

(2) $$m\left(\mathrm{TAA}\right)\left[\%\;\mathrm{of}\;\mathrm{applied}\;\mathrm{amount}\right]=\frac{c\left(\mathrm{TAA}\right)\cdot V\left(\mathrm{acetonitrile}\right)\cdot 15\;\mathrm{mg}\;\cdot 175\;\mathrm{mg}}{m\left(\mathrm{formulation}\right)\cdot m\left(\mathrm{total}\;\mathrm{skin}\;\mathrm{piece}\right)\cdot m(\mathrm{TAA},\;\mathrm{applied})}$$

### 2.7. Incubation of the Skin Samples with Alkanediol Solutions

The same setup as for the ex vivo penetration studies was used for the incubation of the skin samples with the alkanediol solutions. Three skin pieces were incubated with 1 mL of 5% solutions of 2-methyl-2,4-pentanediol, 1,2-pentanediol and 1,2-hexanediol, respectively. Owing to its reduced solubility in water, 1,2-octanediol was applied as a saturated solution. After an incubation period of 24 h, the skin samples were removed from the Franz diffusion cells. The remaining solutions were washed off the skin using cotton swabs soaked with PBS for 30 times. Afterwards, the incubation area was punched out and dabbed dry with cotton swabs.

### 2.8. Preparation of Isolated Stratum Corneum (SC)

The SC was isolated according to a method described by Kligman and Christophers [33] and Zhang and Lunter [34], which does not to affect the lamellar lipid organization of the SC lipids [35]. The skin was placed dermal side down on a filter paper soaked with 0.2% trypsin solution. After 24 h, the digested material was removed with two blunt forceps. The isolated SC was immersed in a 0.05% trypsin inhibitor solution for one minute before being washed five times in succession with highly purified water. The SC sheets obtained were picked up on glass slides and dried over silica gel in a desiccator for at least 72 h.

### 2.9. Confocal Raman Spectroscopy (CRS) Measurements

Measurements with a confocal Raman spectrometer (CRS) were performed using a method described by Liu and Lunter [36,37,38] and Zhang and Lunter [34] to analyze the impact of alkanediols on the SC lipids and the SC thickness. The SC sheets, dried for at least 72 h, were applied to the scanning table of the Raman microscope (alpha 500 R confocal Raman microscope, WITec GmbH, Ulm, Germany). The CRS device was equipped with a 532 nm excitation laser, UHTS (ultra-high throughput) 300 spectrometer and a DV401-BV CCD (charge coupled device) camera. The laser power was set to 10.00 mW with an optical power meter (PM100D, Thorlabs GmbH, Dachau, Germany) before starting the measurements to avoid skin damage due to burning. A 100× objective with a numerical aperture of 0.9 (EC Epiplan-neofluor, Carl Zeiss, Jena, Germany) was used for focusing the excitation radiation onto the skin samples. The backscattered light from the skin samples was spectrally analyzed by an optical grating (600 g/mm) of the spectrometer and was then projected onto the detector of the CRS. The detector was cooled down to −60 °C in advance. The spectra were collected with an integration time of 4 s and 2 accumulations. 

Three SC sheets were measured for each alkanediol solution, and spectra were recorded at three random locations for each SC sheet. This resulted in a total of nine measurements for each solution.

#### 2.9.1. Determination of the Skin Thickness

For determination of the SC thickness, the keratin signal (ν(CH_3_), 2920–2960 cm^−1^) was used. The focus point was moved from 15 µm under a SC sheet to 15 µm above with a step size of 1 µm. The area under the curve (AUC) of the keratin peak was calculated and plotted against the depth. The full width at half maximum (FWHM) of this peak served as skin sample thickness [36,37]. 

When using a dry objective, it was observed that the determined SC thicknesses are generally too low compared to the real thicknesses [37]. Native SC contains water. This means that the corneocytes are filled with water and therefore swollen, and thus more voluminous than in the dried SC. Hence, a higher thickness of native SC compared to dried SC is a consequence of the different water content [39]. However, it was also shown that the used CRS configurations result only in an overall decrease in the measured thickness values [37]. When referring the test samples to a reference sample, the thickness variations caused can be eliminated. Consequently, when comparing test samples to a reference, the conclusions are still correct.

#### 2.9.2. Analysis of Lipid Content

**Figure 1:**
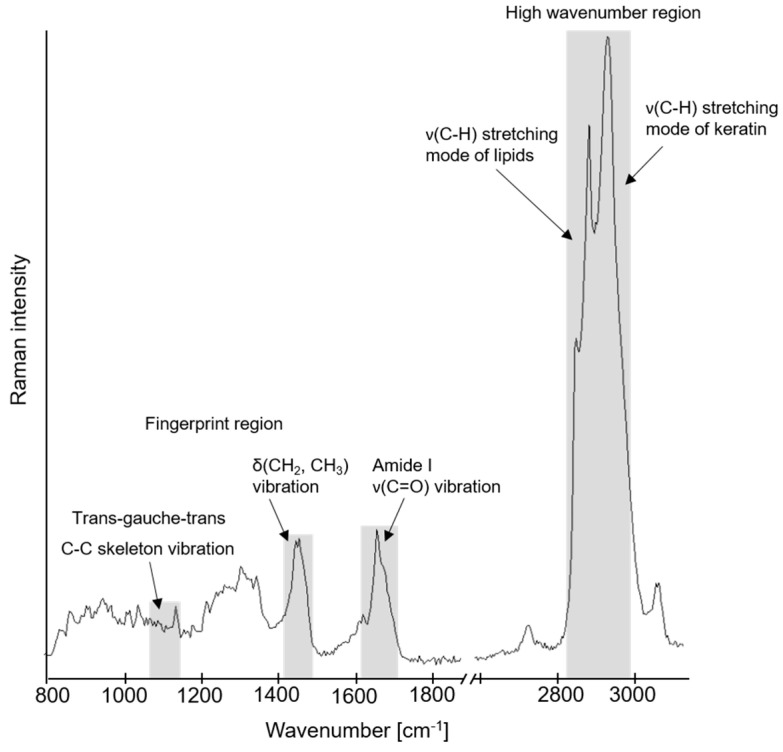
Typical confocal Raman spectroscopy spectrum of stratum corneum with highlighted peaks used for calculation of lipid content and order.

Figure 1 represents a typical CRS spectrum of an SC sheet.

In the fingerprint region of the Raman spectrum of the SC samples, the peak from 1425 to 1490 cm^−1^ belongs to δ(CH_2_, CH_3_)-vibration mode and the one ranging from 1630 to 1710 cm^−1^ to ν(C=O)-mode for Amide I. The latter shows the lowest variation within different measurements of one donor pig as well as between various SC samples originating from different donor pigs. For this reason, this peak is used for normalization [34]. 

The δ(CH_2_, CH_3_) signal 1425–1490 cm^−1^ arises from lipids and proteins and is considered as lipid peak [34,40]. It is used for calculating the lipid amount, related to the Amide I peak according to Equation (3).

(3) $$\mathrm{Normalized}\;\mathrm{signal}\;(\mathrm{fingerprint}\;\mathrm{region})=\frac{{\mathrm{AUC}}_{1425\text{–}1490}}{{\mathrm{AUC}}_{1630\text{–}1710}}$$

In addition, the peaks in the high wavenumber region were examined to obtain further information about the lipid content. In this region the signals originating from keratin and those caused by lipids overlap. Therefore, a Gaussian deconvolution is necessary to separate the content of these peaks. The deconvolution was performed as described in Liu and Lunter [36]. The peaks between 2930 cm^−1^ and 2980 cm^−1^ arise from keratin, whereas the peaks in the range of 2850 cm^−1^ to 2880 cm^−1^ derive from the ν(C-H) symmetric and ν(C-H) asymmetric stretching modes of the lipids, respectively. As in the fingerprint region, these keratin peaks are also employed for normalizing the lipid peaks and determining the lipid content [36,41]. The normalized lipid signal is calculated according to Equation (4).

(4) $$\mathrm{Normalized}\;\mathrm{signal}\;(\mathrm{high}\;\mathrm{wavenumber}\;\mathrm{region})=\frac{{\mathrm{AUC}}_{2850}{+\mathrm{AUC}}_{2880}}{{\mathrm{AUC}}_{2930}{+\mathrm{AUC}}_{2980}}$$

The calculated results of the normalized signals can be used to identify variations of the SC lipid content.

#### 2.9.3. Analysis of Lipid Order

In the fingerprint region, three peaks are associated with the C-C skeleton vibration mode of the long-chained hydrocarbons. These modes are located at wavenumbers of 1060 cm^−1^, 1080 cm^−1^ and 1130 cm^−1^. The peaks at 1060 cm^−1^ and 1130 cm^−1^ correspond to the all-trans conformation which represents the more ordered state of the lipids. The peak at 1080 cm^−1^ corresponds to the gauche conformation and represents the more disordered state of the lipids [42,43,44]. When the lipids are converted from the ordered to the disordered state, the signal of the peak at 1060 cm^−1^ becomes weaker, while the shape of the peak at 1080 cm^−1^ gets broader [42]. 

For reducing the noise and for augmenting the signal-to-noise ratio of the obtained result, a principal component analysis and a polynomial background subtraction are necessary [36]. Moreover, the peak at 1130 cm^−1^ contains part of the weak keratin peak at 1125 cm^−1^ [36,42]. As a result, an adequate integration area has to be selected in order to eliminate the influence of this keratin contribution [36].

The conformational order was calculated using Equation (5) [36,42,44].

(5) $$\mathrm{Conformational}\;\mathrm{order}\;(\mathrm{fingerprint}\;\mathrm{region})=\frac{{\mathrm{AUC}}_{1080}}{{\mathrm{AUC}}_{1060}{+\mathrm{AUC}}_{1130}}$$

Accordingly, a high value of the conformational order represents a predominant gauche conformational order (less-ordered lateral packing of lipids), while a low value is indicative of a trans conformational order (higher-ordered lateral packing of lipids).

### 2.10. Statistical Analysis

The following graphs represent the arithmetic mean ± standard deviation (mean ± SD) extracted from triplicate measurements of the ex vivo skin penetration experiments, or the spectral data obtained from nine independent measurements, respectively. Statistical differences in the total TAA amount in all skin layers as well as differences in the data obtained from CRS measurements were analyzed with a one-way ANOVA (analysis of variance) followed by Tukey’s multiple comparisons test. To consider significant differences in the amount of TAA penetrated into each skin layer, a two-way ANOVA followed by Tukey’s multiple comparisons test was performed. The significant differences are presented with a different number of asterisks (*) as follows: * *p* ≤ 0.05; ** *p* ≤ 0.01; *** *p* ≤ 0.001; **** *p* ≤ 0.0001. Only the differences related to the reference formulations are marked with asterisks in the graphs for better clarity. The statistical significances between the formulations with alkanediols are displayed in the supplementary material.

## 3. Results

### 3.1. Skin Permeation

In all penetration experiments performed the acceptor fluid has been analyzed for permeated TAA. In all cases the concentration of TAA in the acceptor medium was below the LOQ of the HPLC method. This has been expected, since TAA is a highly lipophilic drug (logP 2.53 [45]) and its permeation through the skin is reduced compared to hydrophilic APIs [46,47,48]. Moreover, it is known from previous studies that TAA accumulates in the SC forming a reservoir [49].

### 3.2. Skin Penetration

**Figure 2:**
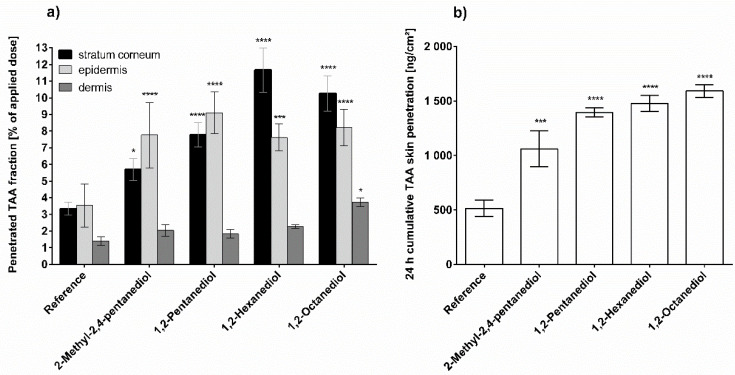
Triamcinolone acetonide penetration from pure aqueous carbomer gel (reference) and aqueous carbomer gel with 5% alkanediol after 24 h incubation; (**a**) Penetrated fraction in stratum corneum, viable epidermis and dermis; (**b**) Cumulative skin penetration of triamcinolone acetonide, *n* = 3, mean ± SD, * *p* ≤ 0.05; *** *p* ≤ 0.001; **** *p* ≤ 0.0001.

Figure 2 represents the results of the TAA penetration experiments from the various ACG formulation variants. The data reveals that the addition of alkanediols significantly increases TAA penetration. Moreover, there is a clear trend that the penetration rates increase with increasing alkyl chain length of the added alkanediol, with 1,2-octanediol showing the most pronounced penetration enhancement. Further, in these samples a significantly increased TAA penetration is also detected in the dermis, whereas the other alkanediols only significantly increased the TAA amount in SC and epidermis. Despite the differences in detail, it is evident from Figure 2 that all alkanediols studied lead to an increased TAA penetration.

**Figure 3:**
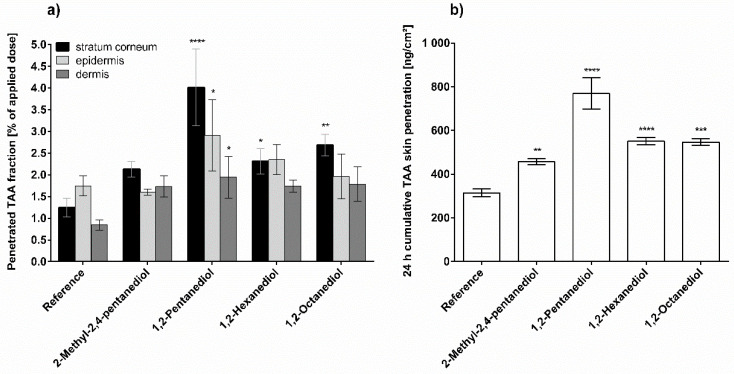
Triamcinolone acetonide penetration from pure anionic hydrophilic cream (reference) and anionic hydrophilic cream with 5% alkanediol after 24 h incubation; (**a**) Penetrated fraction in stratum corneum, viable epidermis and dermis; (**b**) Cumulative skin penetration of triamcinolone acetonide, *n* = 3, mean ± SD, * *p* ≤ 0.05; ** *p* ≤ 0.01; *** *p* ≤ 0.001; **** *p* ≤ 0.0001.

The impact of alkanediols on TAA penetration from AHC is summarized in Figure 3. As can be seen in Figure 3a, 2-methyl-2,4-pentanediol does not significantly vary the TAA penetration within the different skin layers. However, the impact on the total skin penetration is significantly higher compared to the referenced formulation as a consequence of the different calculation methods applied in Figure 3a,b. 1,2-Pentanediol shows the highest penetration rates when considering the three skin layers individually as well as when considering penetration in the whole skin. Both, 1,2-hexanediol and 1,2-octanediol increase the TAA penetration from AHC to a similar extend. The numerical values are intermediate between the ones of 2-methyl-2,4-pentanediol and that of 1,2-pentanediol.

**Figure 4:**
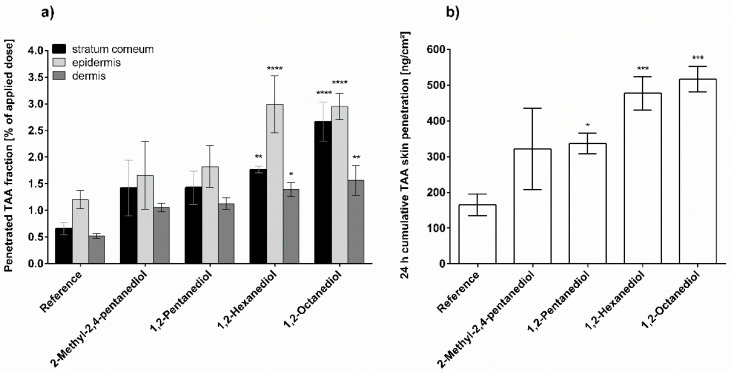
Triamcinolone acetonide penetration from pure nonionic hydrophilic cream (reference) and nonionic hydrophilic cream with 5% alkanediol after 24 h incubation; (**a**) Penetrated fraction in stratum corneum, viable epidermis and dermis; (**b**) Cumulative skin penetration of triamcinolone acetonide, *n* = 3, mean ± SD, * *p* ≤ 0.05; ** *p* ≤ 0.01; *** *p* ≤ 0.001; **** *p* ≤ 0.0001.

Figure 4 depicts the effect of alkanediol addition on TAA penetration from NHC. It can be clearly seen that the longer-chained alkanediols, 1,2-hexanediol and 1,2-octanediol, significantly increase both the TAA fraction penetrated in the different skin layers as well as the cumulative skin penetration expressed as absolute amount of TAA present in the skin after 24 h. The addition of 2-methyl-2,4-pentanediol does not result in a significantly larger penetration. However, this finding seems to be mostly due to the larger standard deviation. Further, 1,2-pentanediol shows an increased cumulative TAA skin penetration. However, the effect is significantly less pronounced compared to the longer-chained alkanediols. Additionally, in contrast to Figure 2a and Figure 3a, the TAA distribution of the reference is reproduced in the NHC containing the investigated alkanediols. The TAA concentration is highest in the viable epidermis in all samples investigated.

**Figure 5:**
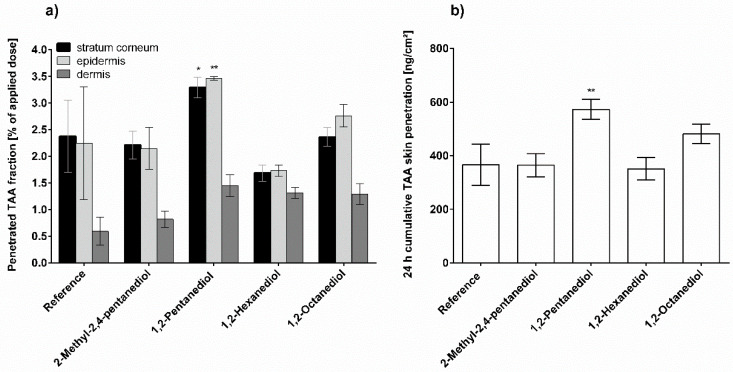
Triamcinolone acetonide penetration from pure basic cream (reference, containing 10% propylene glycol) and basic cream with 5% alkanediol after 24 h incubation; (**a**) Penetrated fraction in stratum corneum, viable epidermis and dermis; (**b**) Cumulative skin penetration of triamcinolone acetonide, *n* = 3, mean ± SD, * *p* ≤ 0.05; ** *p* ≤ 0.01.

As can be taken from Figure 5, generally, the effect of alkanediols on the TAA penetration from BC is small. 1,2-Pentanediol is the only alkanediol that significantly increases the penetration of TAA. All other alkanediols do not enhance the TAA penetration. It is worth mentioning, that the compendial BC reference formulation contains 10% propylene glycol, which was replaced by 5% of the respective alkanediols to obtain the test formulations. The remaining 5% were supplemented with water. As propylene glycol itself is also known to act as penetration enhancer [50], the impact of the added alkanediols on TAA penetration is expectedly low. Only 1,2-pentanediol shows a small but significant enhanced penetration compared to the reference formulation containing propylene glycol.

### 3.3. Confoal Raman Spectroscopy Measurements

#### 3.3.1. Lipid Content and Stratum Corneum Thickness

The amphiphilic nature of alkanediols enables them to interact with the microbial cell membranes [51] as well as with the surfactant and co-surfactant system of the cream bases used [4]. Thus, it appeared very likely that they also interact with SC lipids. To investigate this interaction, CRS measurements were conducted.

**Figure 6:**
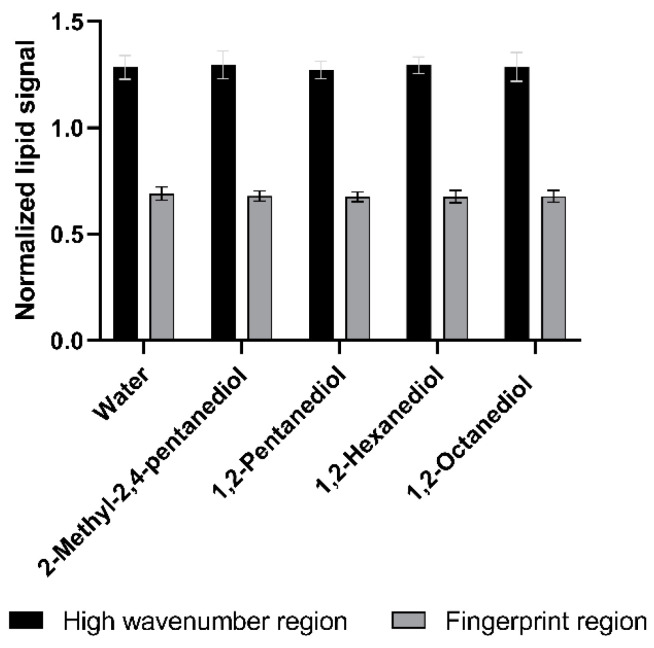
Normalized lipid signals in high wavenumber and fingerprint regions for lipid content analysis, after treatment with water and 5% solutions of 2-methyl-2,4-pentanediol, 1,2-pentanediol and 1,2-hexanediol, and saturated 1,2-octanediol solution, *n* = 9, mean ± SD.

Figure 6 depicts the normalized lipid signals which represent the lipid content of the SC after 24 h incubation with the respective alkanediol solutions in comparison to pure water.

From these results it is obvious, that the treatment with alkanediols does not alter the SC lipid content in comparison to incubation with water. The results for the lipid content analysis in the high wavenumber and fingerprint regions are identical. This indicates that the tested alkanediols are not able to extract lipids from the SC.

**Figure 7:**
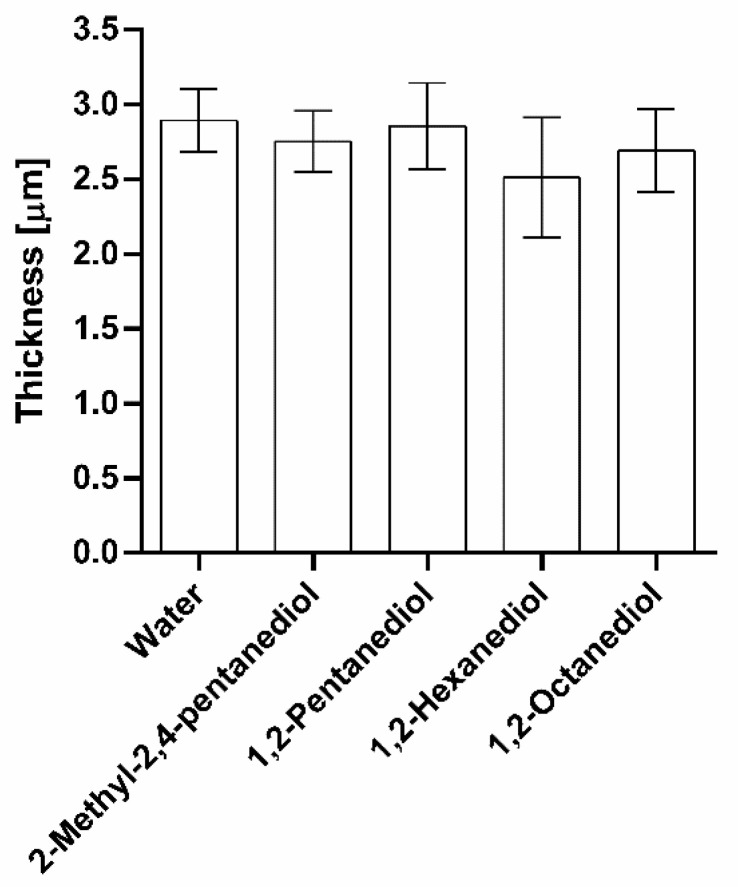
Measured thicknesses of SC after treatment with water and 5% solutions of 2-methyl-2,4-pentanediol, 1,2-pentanediol and 1,2-hexanediol, and saturated 1,2-octanediol solution, *n* = 9, mean ± SD.

As already described above, the thicknesses measured using this CRS methodology do not correspond to the “real” thicknesses of the SC. The measured values are all systematically lower than the values measured on SC sheets not prepared as necessary for this method. However, despite this systematic deviation, conclusions are valid as far as the results are seen in relation to a respective reference sample, i.e., water treated specimen. It can be clearly taken from Figure 7 that all measured thicknesses after alkanediol treatment do not differ significantly from the water treated samples. Thus, it can be concluded that the alkanediols do not have an impact on the SC thickness. These findings are in good agreement with the results for the lipid content analysis. As thinning of the SC is usually linked to lipid extraction [34] and as no lipids were extracted by the alkanediols, the SC thicknesses do not shrink either.

#### 3.3.2. Lipid Order

**Figure 8:**
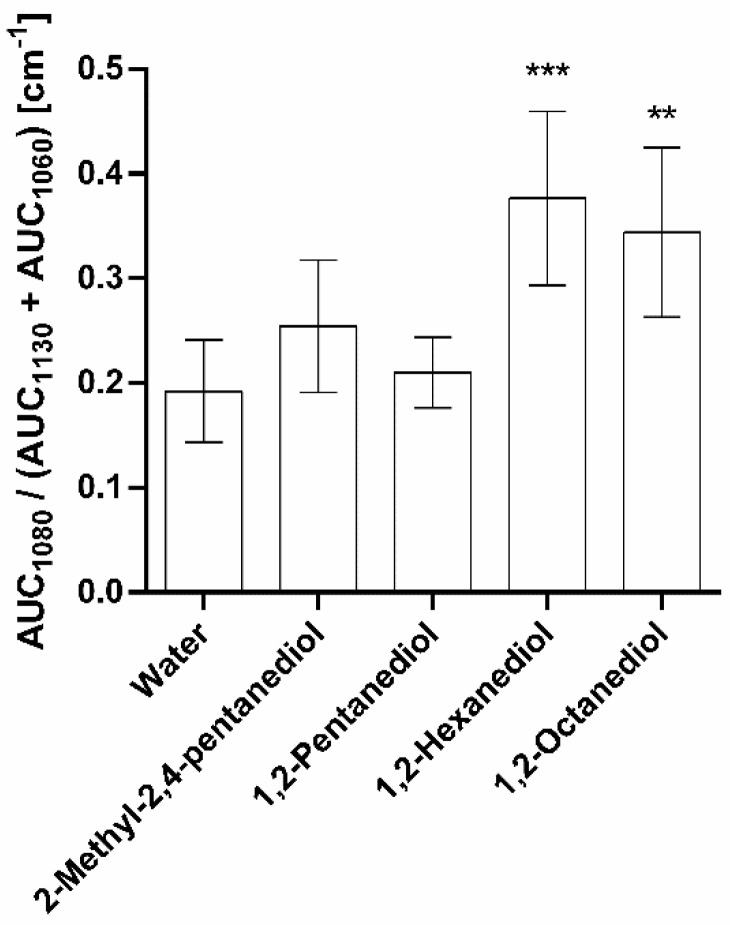
C-C skeleton vibration mode: calculation of ratio for lipid order analysis, after treatment with water and 5% solutions of 2-methyl-2,4-pentanediol, 1,2-pentanediol and 1,2-hexanediol, and saturated 1,2-octanediol solution, *n* = 9, mean ± SD, ** *p* ≤ 0.01; *** *p* ≤ 0.001.

Figure 8 presents the alteration of SC lipid order caused by the alkanediol solutions. The peak at 1080 cm^−1^ represents the gauche conformation whereas the peaks at 1060 cm^−1^ and 1130 cm^−1^ correspond to the all-trans conformation of the lipids. A rising ratio of these signals indicates an increase in the gauche fraction representing a more disordered state of the SC lipids.

1,2-Pentanediol and 2-methyl-2,4-pentanediol do not show a significant effect on the lipid order compared to the water reference. In contrast, the longer-chained alkanediols, 1,2-hexanediol and 1,2-octanediol lead to a significant rise in this ratio, indicating a less-ordered state of the lipids. Thus, the alteration of lipid order depends on the alkyl chain length of the alkanediol applied. The shorter-chained alkanediols do not seem to be able to intercalate into the SC lipids, whereas the alkyl chain of the longer-chained ones seems to be long enough for an intercalation. As a result, the SC lipids become disordered.

## 4. Discussion

The target location of topically applied glucocorticoids, which is the glucocorticoid receptor, is located in the living skin layers [17]. Therefore, when applying the TAA-containing formulations, TAA has to overcome the SC, forming the main entrance barrier, before being able to reach its site of action in the deeper skin layers.

Release from the semisolid preparation is the first step for the transport of an API into the skin. Detailed TAA release experiments from the same creams used in the present study revealed a general decrease when adding alkanediols. In contrast, TAA release increased when alkanediols were added to the ACG hydrogel [18]. This underlines as already known from diverse research [52,53,54] that API release from a dermal formulation is an important quality factor but does only partially describe the biopharmaceutical properties of a dermal preparation.

The presented results reveal that alkanediols affect TAA penetration in various ways depending on the base formulation considered. 

In general, the penetration of a drug can be enhanced by increasing its thermodynamic activity in the vehicle. As known from previous studies, 0.1% TAA is above its saturation in all formulations studied yielding a suspension of undissolved TAA in a saturated vehicle [18]. Accordingly, the thermodynamic activity of TAA in all tested preparation equals 1. Consequently, a larger or smaller fraction of undissolved TAA does not alter its thermodynamic activity and thus cannot be the cause for the observed penetration enhancement [55,56].

Thus, it seems to be more likely that the increased penetration of TAA in the presence of alkanediols is due to an increased solubility of the drug in the SC. Alkanediol molecules are known to penetrate the SC and increase the overall API solubility in the skin barrier [57,58]. This would shift the TAA distribution towards the SC. Therefore, the respective solubility parameters of TAA, the alkanediols and the SC are considered and summarized in Table 2. The solubility parameters δ allow to make a theoretical prediction of the solubility of compounds in one another. In the present study, these parameters predict the solubility of TAA in the alkanediols and in the SC. The lower the difference in δ-values between TAA and a substance, the higher the solubility of TAA in the corresponding substance [59]. Correspondingly, the solubility of TAA increases when Δδ approximates zero

As can be taken from Table 2, the difference in the solubility parameters between TAA and the SC is very low (1.71 (MPa)^½^), indicating a high affinity of TAA for this skin layer [63]. This is associated with TAA accumulation and consequently a relatively high fraction of TAA can be found in the SC in all skin samples even after 24 h incubation. Thus, TAA forms a reservoir in the outermost skin layers from which it is released over a prolonged time period exhibiting a depot effect [49]. This result is consistent with the fact that no TAA could be detected in the acceptor medium.

The solubility parameters of the alkanediols and TAA are similar. However, the ∆δ values between TAA and the alkanediols are larger than the difference of the δ values between TAA and SC. Since the solubility of TAA in the SC is already very high, the alkanediols are not expected to have a further significant effect on this factor [64]. Therefore, no effects of the alkanediols can be deduced merely by considering the solubility parameters.

However, it must be considered that the SC is not a homogeneous membrane as it contains both lipophilic and hydrophilic regions. Propylene glycol is known to be inserted into the latter [65,66]. Therefore, it could be hypothesized that 1,2-pentanediol and 2-methyl-2,4-pentanediol are also incorporated into the hydrophilic regions of the lamellar structured SC lipids. As mentioned previously, an earlier study revealed that the release from the tested creams is reduced by the addition of alkanediols [18] while the penetration is increased. Therefore, it is likely that, on the one hand, the alkanediols interact with the SC lipids. On the other hand, as described for propylene glycol, solvation of the keratin of the corneocytes might attribute to the penetration enhancement of the shorter-chained alkanediols [67].

Moreover, some studies suggest that for propylene glycol a solvent drag effect is also responsible for an enhanced drug penetration [50,68]. Additionally, 1,2-pentanediol is suspected to act as a kind of a carrier for the dissolved drug [11]. Lee et al. [51] found alkanediols to be absorbed by the skin. This percutaneous absorption reduces with increasing alkyl chain length. Consequently, 1,2-pentanediol shows the highest penetration [51]. This might indicate that a solvent drag effect additionally contributes to the larger TAA penetration from the 1,2-pentanediol containing formulations.

As alkanediols proved to be surface active, they are predestined to interact with the SC lipids. This alters the barrier function of the SC and consequently affects skin penetration [55]. Therefore, the influence of the tested alkanediols on the content and ordering of SC lipids was examined by means of CRS. While these experiments did not reveal any lipid loss, it became evident that alkanediols increasingly disrupt the lipid order when the alkyl chain length of the alkanediols rises. 

Warner et al. found for alkanediols that when exceeding a certain alkyl chain length, a further increase in the number of methylene groups does no longer contribute to an enhanced disordering of SC lipids. 1,2-Hexanediol and 1,2-octanediol intercalate into the lipid bilayer to the same depth [69]. This is in concordance with our results, showing no significant difference between the gauche/trans ratio of 1,2-hexanediol and 1,2-octanediol. However, it should be noted that the aqueous solubility of 1,2-octanediol is 1.82% [70] and its density is lower than that of water. As a result, the 1,2-octanediol excess floats on top of the incubation solution. Therefore, although 5% 1,2-octanediol have been applied the skin is only in contact with a saturated solution. Thus, the actual concentration applied to the skin is lower than that of the other alkanediols, which dissolve completely in water at a concentration of 5%. Obviously that lower concentration of 1,2-octanediol is still high enough for the intercalation and disordering of SC lipids equally effective as 5% 1,2-hexanediol.

These results indicate that the increased TAA penetration from the formulations with incorporated 1,2-hexanediol and 1,2-octanediol can be explained by disordering the SC lipids. This results in a reduced barrier function of the SC allowing the API to penetrate the SC more easily. As the shorter-chained alkanediols 1,2-pentanediol and 2-methyl-2,4-pentanediol do not substantially affect the lipid order, their penetration enhancement must originate from the effects discussed above.

Differently from the other results, 2-methyl-2,4-pentanediol does not show such a strong penetration-enhancing effect in comparison to the other alkanediols. This alkanediol does not have vicinal hydroxyl groups and therefore has a lower amphiphilicity (data not shown). As a result, 2-methyl-2,4-pentanediol differs in its properties from the other alkanediols studied. Since the extent of antimicrobial activity of the alkanediols depends on their amphiphilicity, which favors their incorporation into the lipophilic bilayer of the bacterial cell wall, 2-methyl-2,4-pentanediol was found to be less effective in antimicrobial performance than the other alkanediols. In addition, due to the reduced amphiphilicity, the interaction with surfactants and co-surfactants present in the formulation is likewise diminished [4]. In our study, the reduced amphiphilicity leads, on the one hand, to a lower TAA solubility of this compound compared to the other alkanediols [18]. On the other hand, the interaction with the SC components is sterically less favorable.

When comparing the different test formulations, it is noticeable that the penetrated TAA amounts from the gels are generally the highest. For the final evaluation it must be considered that the different formulations had to be assessed using skin samples originating from different donor pigs, as one porcine ear delivers only a limited number of skin punches. However, TAA penetration from the hydrogels is almost double compared to the creams. Moreover, in contrast to the creams TAA release from the hydrogels was much faster than from those [18]. As the penetration experiments were performed under finite, non-occlusive conditions, it seems, thus, to be more likely that the comparatively high penetration rate from the hydrogels is associated with the evaporation of water. This causes the TAA concentration on the skin to increase and consequently enhance penetration [71]. Since the hydrogels consist mainly of water, and TAA can be present only in the aqueous phase this effect is here much more pronounced than in the creams.

## 5. Conclusions

In this study, the influence of four different alkanediols on skin penetration of TAA from different semisolid formulations was investigated. The results revealed that the alkanediols tested generally act as penetration enhancers. However, the magnitude of this effect differs depending on the formulation used.

CRS measurements revealed that the studied alkanediols did not lead to an extraction of lipids from SC and consequently the SC thickness was also not altered. However, SC lipids enter a more disordered state as a result of the incubation with the longer-chained alkanediols. This can explain the enhanced TAA penetration when these longer-chained alkanediols are added. In addition, a solvent drag effect, as well as an interaction with the hydrophilic regions of the lamellar ordered SC lipids or the keratin of corneocytes, provides an explanation for the increased penetration from the formulations containing the shorter-chained alkanediols. 

2-Methyl-2,4-pentanediol showed only a marginal effect on TAA penetration. This could have been expected as it is not a 1,2-alkanediol. Hence, it has a lower amphiphilicity and its interaction with the SC lipids as well as cell membranes [4] is comparatively lower than those of the other alkanediols.

Overall, alkanediols increased TAA penetration from the formulations tested to a more or less pronounced extent. This should be considered when substituting alkanediols for conventional antimicrobial preservatives. The penetration-enhancing effect depends on both, the respective formulation and the specific alkanediol added. To this end it is unfortunate that it seems almost impossible to predict anything quantitatively due to the extremely high colloidal complexity of the total system comprising cream and skin. Partitioning and solubility of TAA and alkanediols in the diverse compartments/phases of the cream and the skin affects each other and alters also the structural integrity of the preparations. This might then likewise affect the physical stability and the consistency of the respective formulation as has been shown earlier [4].

## Figures and Tables

**Table 1:** Composition (in % *w*/*w*) of the compendial bases without TAA and alkanediols.

(a) ACG		(b) AHC		(c) NHC		(d) BC	
Carbomer 50.000	0.5	Emulsifying cetostearyl alcohol type A	9.0	Polysorbate 60	5.0	Glycerol mono-stearate 60	4.0
Sodium hydroxide solution 5%	3.0	Liquid paraffin	10.5	Cetostearyl alcohol	10.0	Cetyl alcohol	6.0
Purified water	96.5	White soft paraffin	10.5	White soft paraffin	25.0	Medium-chain triglycerides	7.5
		Purified water	70.0	Glycerol 85%	10.0	White soft paraffin	25.5
				Purified water	50.0	Macrogol 20 glycerol monostearate	7.0
						Propylene glycol	10.0
						Purified water	40.0

ACG: aqueous carbomer gel, AHC: anionic hydrophilic cream, NHC: nonionic hydrophilic cream, BC: basic cream.

**Table 2:** Solubility parameters (δ) of the substances and differences of solubility parameters between the alkanediols, stratum corneum and triamcinolone acetonide (∆δ).

	δ [(MPa)^½^]	∆δ [(MPa)^½^]
Triamcinolone acetonide	20.10 ^a^	-
2-Methyl-2,4-pentanediol	26.79 ^b^	6.66
1,2-Pentanediol	24.98 ^b^	4.88
1,2-Hexanediol	24.13 ^b^	4.03
1,2-Octanediol	22.92 ^b^	2.82
Stratum corneum	21.81 ^c,d^	1.71

^a^ Abou-ElNour et al. [60]; ^b^ Sigg and Daniels [18]; ^c^ Ezati et al. [61], ^d^ Venkatram et al. [62].

## Data Availability

Not applicable.

## Supplementary Materials

The following are available online at https://www.mdpi.com/article/10.3390/pharmaceutics13091451/s1.
