# Supplementary material for: Impact of Alkanediols on Stratum Corneum Lipids and Triamcinolone Acetonide Skin Penetration
Source: Pharmaceutics. 2021 Sep 11;13(9):1451. doi: 10.3390/pharmaceutics13091451 (PMC8469070; doi:10.3390/pharmaceutics13091451)
Supplement: Supplementary file 1 [file pharmaceutics-13-01451-s001.zip › pharmaceutics-1317008-SI.pdf]

pharmaceutics

Supplementary Materials: Impact of Alkanediols on Stratum Corneum Lipids and Triamcinolone Acetonide Skin Penetration

Melanie Sigg, Rolf Daniels

Statistical Analysis of TAA skin penetration

Penetrated fraction in stratum corneum, viable epidermis and dermis:
two-way ANOVA followed by Tukey's multiple comparisons test
Cumulative skin penetration of triamcinolone acetonide:
one-way ANOVA followed by Tukey's multiple comparisons test
The significant differences are presented with a different number of asterisks (*) as follows: *
$p \leq 0.05$; ** $p \leq 0.01$; *** $p \leq 0.001$; **** $p \leq 0.0001$. ns: not significant.

Aqueous carbomer gel

a) Penetrated fraction in stratum corneum, viable epidermis and dermis

Stratum corneum

Reference vs. 2-Methyl-2,4-pentanediol 5 % *
Reference vs. 1,2-Pentanediol 5 % ****
Reference vs. 1,2-Hexanediol 5 % ****
Reference vs. 1,2-Octanediol 5 % ****
2-Methyl-2,4-pentanediol 5 % vs. 1,2-Pentanediol 5 % ns
2-Methyl-2,4-pentanediol 5 % vs. 1,2-Hexanediol 5 % ****
2-Methyl-2,4-pentanediol 5 % vs. 1,2-Octanediol 5 % ****
1,2-Pentanediol 5 % vs. 1,2-Hexanediol 5 % ***
1,2-Pentanediol 5 % vs. 1,2-Octanediol 5 % *
1,2-Hexanediol 5 % vs. 1,2-Octanediol 5 % ns

Epidermis

Reference vs. 2-Methyl-2,4-pentanediol 5 % ****
Reference vs. 1,2-Pentanediol 5 % ****
Reference vs. 1,2-Hexanediol 5 % ****
Reference vs. 1,2-Octanediol 5 % ****
2-Methyl-2,4-pentanediol 5 % vs. 1,2-Pentanediol 5 % ns
2-Methyl-2,4-pentanediol 5 % vs. 1,2-Hexanediol 5 % ns
2-Methyl-2,4-pentanediol 5 % vs. 1,2-Octanediol 5 % ns
1,2-Pentanediol 5 % vs. 1,2-Hexanediol 5 % ns
1,2-Pentanediol 5 % vs. 1,2-Octanediol 5 % ns
1,2-Hexanediol 5 % vs. 1,2-Octanediol 5 % ns

Dermis

Reference vs. 2-Methyl-2,4-pentanediol 5 % ns
Reference vs. 1,2-Pentanediol 5 % ns
Reference vs. 1,2-Hexanediol 5 % ns
Reference vs. 1,2-Octanediol 5 % *
2-Methyl-2,4-pentanediol 5 % vs. 1,2-Pentanediol 5 % ns
2-Methyl-2,4-pentanediol 5 % vs. 1,2-Hexanediol 5 % ns
2-Methyl-2,4-pentanediol 5 % vs. 1,2-Octanediol 5 % ns
1,2-Pentanediol 5 % vs. 1,2-Hexanediol 5 % ns
1,2-Pentanediol 5 % vs. 1,2-Octanediol 5 % ns
1,2-Hexanediol 5 % vs. 1,2-Octanediol 5 % ns

b) Cumulative skin penetration of triamcinolone acetonide
Reference vs. 2-Methyl-2,4-pentanediol 5 % ***
Reference vs. 1,2-Pentanediol 5 % ****
Reference vs. 1,2-Hexanediol 5 % ****
Reference vs. 1,2-Octanediol 5 % ****
2-Methyl-2,4-pentanediol 5 % vs. 1,2-Pentanediol 5 % **
2-Methyl-2,4-pentanediol 5 % vs. 1,2-Hexanediol 5 % **
2-Methyl-2,4-pentanediol 5 % vs. 1,2-Octanediol 5 % ***
1,2-Pentanediol 5 % vs. 1,2-Hexanediol 5 % ns
1,2-Pentanediol 5 % vs. 1,2-Octanediol 5 % ns
1,2-Hexanediol 5 % vs. 1,2-Octanediol 5 % ns

Anionic hydrophilic cream

a) Penetrated fraction in stratum corneum, viable epidermis and dermis

Stratum corneum

Reference vs. 2-Methyl-2,4-pentanediol 5 % ns
Reference vs. 1,2-Pentanediol 5 % ****
Reference vs. 1,2-Hexanediol 5 % *
Reference vs. 1,2-Octanediol 5 % **
2-Methyl-2,4-pentanediol 5 % vs. 1,2-Pentanediol 5 % ****
2-Methyl-2,4-pentanediol 5 % vs. 1,2-Hexanediol 5 % ns
2-Methyl-2,4-pentanediol 5 % vs. 1,2-Octanediol 5 % ns
1,2-Pentanediol 5 % vs. 1,2-Hexanediol 5 % ***
1,2-Pentanediol 5 % vs. 1,2-Octanediol 5 % **
1,2-Hexanediol 5 % vs. 1,2-Octanediol 5 % ns

Epidermis

Reference vs. 2-Methyl-2,4-pentanediol 5 % ns
Reference vs. 1,2-Pentanediol 5 % *
Reference vs. 1,2-Hexanediol 5 % ns
Reference vs. 1,2-Octanediol 5 % ns
2-Methyl-2,4-pentanediol 5 % vs. 1,2-Pentanediol 5 % **
2-Methyl-2,4-pentanediol 5 % vs. 1,2-Hexanediol 5 % ns
2-Methyl-2,4-pentanediol 5 % vs. 1,2-Octanediol 5 % ns
1,2-Pentanediol 5 % vs. 1,2-Hexanediol 5 % ns
1,2-Pentanediol 5 % vs. 1,2-Octanediol 5 % ns
1,2-Hexanediol 5 % vs. 1,2-Octanediol 5 % ns

Dermis

Reference vs. 2-Methyl-2,4-pentanediol 5 % ns
Reference vs. 1,2-Pentanediol 5 % *
Reference vs. 1,2-Hexanediol 5 % ns
Reference vs. 1,2-Octanediol 5 % ns
2-Methyl-2,4-pentanediol 5 % vs. 1,2-Pentanediol 5 % ns
2-Methyl-2,4-pentanediol 5 % vs. 1,2-Hexanediol 5 % ns
2-Methyl-2,4-pentanediol 5 % vs. 1,2-Octanediol 5 % ns
1,2-Pentanediol 5 % vs. 1,2-Hexanediol 5 % ns
1,2-Pentanediol 5 % vs. 1,2-Octanediol 5 % ns
1,2-Hexanediol 5 % vs. 1,2-Octanediol 5 % ns
b) Cumulative skin penetration of triamcinolone acetonide
Reference vs. 2-Methyl-2,4-pentanediol 5 % **
Reference vs. 1,2-Pentanediol 5 % ****

Reference vs. 1,2-Hexanediol 5 % ****
Reference vs. 1,2-Octanediol 5 % ****
2-Methyl-2,4-pentanediol 5 % vs. 1,2-Pentanediol 5 % ****
2-Methyl-2,4-pentanediol 5 % vs. 1,2-Hexanediol 5 % *
2-Methyl-2,4-pentanediol 5 % vs. 1,2-Octanediol 5 % ns
1,2-Pentanediol 5 % vs. 1,2-Hexanediol 5 % ***
1,2-Pentanediol 5 % vs. 1,2-Octanediol 5 % ***
1,2-Hexanediol 5 % vs. 1,2-Octanediol 5 % ns

Nonionic hydrophilic cream

a) Penetrated fraction in stratum corneum, viable epidermis and dermis

Stratum corneum

Reference vs. 2-Methyl-2,4-pentanediol 5 % ns
Reference vs. 1,2-Pentanediol 5 % ns
Reference vs. 1,2-Hexanediol 5 % **
Reference vs. 1,2-Octanediol 5 % ****
2-Methyl-2,4-pentanediol 5 % vs. 1,2-Pentanediol 5 % ns
2-Methyl-2,4-pentanediol 5 % vs. 1,2-Hexanediol 5 % ns
2-Methyl-2,4-pentanediol 5 % vs. 1,2-Octanediol 5 % ***
1,2-Pentanediol 5 % vs. 1,2-Hexanediol 5 % ns
1,2-Pentanediol 5 % vs. 1,2-Octanediol 5 % ***
1,2-Hexanediol 5 % vs. 1,2-Octanediol 5 % *

Epidermis

Reference vs. 2-Methyl-2,4-pentanediol 5 % ns
Reference vs. 1,2-Pentanediol 5 % ns
Reference vs. 1,2-Hexanediol 5 % ****
Reference vs. 1,2-Octanediol 5 % ****
2-Methyl-2,4-pentanediol 5 % vs. 1,2-Pentanediol 5 % ns
2-Methyl-2,4-pentanediol 5 % vs. 1,2-Hexanediol 5 % ***
2-Methyl-2,4-pentanediol 5 % vs. 1,2-Octanediol 5 % ***
1,2-Pentanediol 5 % vs. 1,2-Hexanediol 5 % **
1,2-Pentanediol 5 % vs. 1,2-Octanediol 5 % **
1,2-Hexanediol 5 % vs. 1,2-Octanediol 5 % ns

Dermis

Reference vs. 2-Methyl-2,4-pentanediol 5 % ns
Reference vs. 1,2-Pentanediol 5 % ns
Reference vs. 1,2-Hexanediol 5 % *
Reference vs. 1,2-Octanediol 5 % **
2-Methyl-2,4-pentanediol 5 % vs. 1,2-Pentanediol 5 % ns
2-Methyl-2,4-pentanediol 5 % vs. 1,2-Hexanediol 5 % ns
2-Methyl-2,4-pentanediol 5 % vs. 1,2-Octanediol 5 % ns
1,2-Pentanediol 5 % vs. 1,2-Hexanediol 5 % ns
1,2-Pentanediol 5 % vs. 1,2-Octanediol 5 % ns
1,2-Hexanediol 5 % vs. 1,2-Octanediol 5 % ns
b) Cumulative skin penetration of triamcinolone acetonide
Reference vs. 2-Methyl-2,4-pentanediol 5 % ns
Reference vs. 1,2-Pentanediol 5 % *
Reference vs. 1,2-Hexanediol 5 % ***
Reference vs. 1,2-Octanediol 5 % ***
2-Methyl-2,4-pentanediol 5 % vs. 1,2-Pentanediol 5 % ns

2-Methyl-2,4-pentanediol 5 % vs. 1,2-Hexanediol 5 % ns
2-Methyl-2,4-pentanediol 5 % vs. 1,2-Octanediol 5 % *
1,2-Pentanediol 5 % vs. 1,2-Hexanediol 5 % ns
1,2-Pentanediol 5 % vs. 1,2-Octanediol 5 % *
1,2-Hexanediol 5 % vs. 1,2-Octanediol 5 % ns

Basic cream

a) Penetrated fraction in stratum corneum, viable epidermis and dermis

Stratum corneum

Reference vs. 2-Methyl-2,4-pentanediol 5 % ns
Reference vs. 1,2-Pentanediol 5 % *
Reference vs. 1,2-Hexanediol 5 % ns
Reference vs. 1,2-Octanediol 5 % ns
2-Methyl-2,4-pentanediol 5 % vs. 1,2-Pentanediol 5 % *
2-Methyl-2,4-pentanediol 5 % vs. 1,2-Hexanediol 5 % ns
2-Methyl-2,4-pentanediol 5 % vs. 1,2-Octanediol 5 % ns
1,2-Pentanediol 5 % vs. 1,2-Hexanediol 5 % ***
1,2-Pentanediol 5 % vs. 1,2-Octanediol 5 % *
1,2-Hexanediol 5 % vs. 1,2-Octanediol 5 % ns

Epidermis

Reference vs. 2-Methyl-2,4-pentanediol 5 % ns
Reference vs. 1,2-Pentanediol 5 % **
Reference vs. 1,2-Hexanediol 5 % ns
Reference vs. 1,2-Octanediol 5 % ns
2-Methyl-2,4-pentanediol 5 % vs. 1,2-Pentanediol 5 % **
2-Methyl-2,4-pentanediol 5 % vs. 1,2-Hexanediol 5 % ns
2-Methyl-2,4-pentanediol 5 % vs. 1,2-Octanediol 5 % ns
1,2-Pentanediol 5 % vs. 1,2-Hexanediol 5 % ****
1,2-Pentanediol 5 % vs. 1,2-Octanediol 5 % ns
1,2-Hexanediol 5 % vs. 1,2-Octanediol 5 % *

Dermis

Reference vs. 2-Methyl-2,4-pentanediol 5 % ns
Reference vs. 1,2-Pentanediol 5 % ns
Reference vs. 1,2-Hexanediol 5 % ns
Reference vs. 1,2-Octanediol 5 % ns
2-Methyl-2,4-pentanediol 5 % vs. 1,2-Pentanediol 5 % ns
2-Methyl-2,4-pentanediol 5 % vs. 1,2-Hexanediol 5 % ns
2-Methyl-2,4-pentanediol 5 % vs. 1,2-Octanediol 5 % ns
1,2-Pentanediol 5 % vs. 1,2-Hexanediol 5 % ns
1,2-Pentanediol 5 % vs. 1,2-Octanediol 5 % ns
1,2-Hexanediol 5 % vs. 1,2-Octanediol 5 % ns
b) Cumulative skin penetration of triamcinolone acetonide
Reference vs. 2-Methyl-2,4-pentanediol 5 % ns
Reference vs. 1,2-Pentanediol 5 % **
Reference vs. 1,2-Hexanediol 5 % ns
Reference vs. 1,2-Octanediol 5 % ns
2-Methyl-2,4-pentanediol 5 % vs. 1,2-Pentanediol 5 % **
2-Methyl-2,4-pentanediol 5 % vs. 1,2-Hexanediol 5 % ns
2-Methyl-2,4-pentanediol 5 % vs. 1,2-Octanediol 5 % ns
1. ,2-Pentanediol 5 % vs. 1,2-Hexanediol 5 % **

1. ,2-Pentanediol 5 % vs. 1,2-Octanediol 5 % ns
1. ,2-Hexanediol 5 % vs. 1,2-Octanediol 5 % ns
